# Emotion Recognition from EEG Signals Using Multidimensional Information in EMD Domain

**DOI:** 10.1155/2017/8317357

**Published:** 2017-08-16

**Authors:** Ning Zhuang, Ying Zeng, Li Tong, Chi Zhang, Hanming Zhang, Bin Yan

**Affiliations:** ^1^China National Digital Switching System Engineering and Technological Research Center, Zhengzhou 450002, China; ^2^Key Laboratory for NeuroInformation of Ministry of Education, School of Life Science and Technology, University of Electronic Science and Technology of China, Chengdu, China

## Abstract

This paper introduces a method for feature extraction and emotion recognition based on empirical mode decomposition (EMD). By using EMD, EEG signals are decomposed into Intrinsic Mode Functions (IMFs) automatically. Multidimensional information of IMF is utilized as features, the first difference of time series, the first difference of phase, and the normalized energy. The performance of the proposed method is verified on a publicly available emotional database. The results show that the three features are effective for emotion recognition. The role of each IMF is inquired and we find that high frequency component IMF1 has significant effect on different emotional states detection. The informative electrodes based on EMD strategy are analyzed. In addition, the classification accuracy of the proposed method is compared with several classical techniques, including fractal dimension (FD), sample entropy, differential entropy, and discrete wavelet transform (DWT). Experiment results on DEAP datasets demonstrate that our method can improve emotion recognition performance.

## 1. Introduction

Emotion plays an important role in our daily life and work. Real-time assessment and regulation of emotion will improve people's life and make it better. For example, in the communication of human-machine-interaction, emotion recognition will make the process more easy and natural. Another example, in the treatment of patients, especially those with expression problems, the real emotion state of patients will help doctors to provide more appropriate medical care. In recent years, emotion recognition from EEG has gained mass attention. Also it is a very important factor in brain computer interface (BCI) systems, which will effectively improve the communication between human and machines [1].

Various features and extraction methods have been proposed for emotion recognition from EEG signals, including time domain techniques, frequency domain techniques, joint time-frequency analysis techniques, and other strategies.

Statistics of EEG series, that is, first and second difference, mean value, and power are usually used in time domain [2]. Nonlinear features, including fractal dimension (FD) [3, 4], sample entropy [5], and nonstationary index [6], are utilized for emotion recognition. Hjorth features [7] had also been used in EEG studies [8, 9]. Petrantonakis and Hadjileontiadis introduced higher order crossings (HOC) features to capture the oscillatory pattern of EEG [10]. Wang et al. extracted frequency domain features for classification [11]. Time-frequency analysis is based on the spectrum of EEG signals; then the energy, power, power spectral density (PSD), and differential entropy [12] of certain subband are usually utilized as features. Short-time Fourier transform (STFT) [13, 14], Hilbert-Huang transform (HHT) [15, 16], and discrete wavelet transform (DWT) [17–19] are the most commonly used techniques for spectrum calculating. It has been commonly tested and verified that higher frequency subband such as Beta (16–32 Hz) and Gamma (32–64 Hz) bands outperforms lower subband for emotion recognition [20, 21].

Other features extracted from combination of electrode are utilized too, such as coherence and asymmetry of electrodes in different brain regions [22–24] and graph-theoretic features [25]. Jenke et al. had done a research comparing the performance of different features mentioned above and got a guiding rule for feature extraction and selection [26].

Some other strategies such as utilizing deep network to improve the classification performance have also been researched. Zheng and Lu used deep neural network to investigate critical frequency bands and channels for emotion recognition [27]. Yang et al. used hierarchical network with subnetwork nodes for emotion recognition [28].

EMD is proposed by Huang et al. in 1998 [29]. Unlike DWT, which needs to predetermine transform base function and decomposition level, EMD can decompose signals into IMF automatically. These IMFs represent different frequency components of original signals, with band-limited characteristic. By applying Hilbert transform to IMF, we can get instantaneous phase information of IMF. So EMD is suitable for analysis of nonlinear and nonstationary sequence, such as neural signals.

EMD is a good choice for EEG signals and we utilize it for emotion recognition from EEG data. Which feature is effective for emotion recognition in EMD domain? Which IMF component is best for classification? Is the performance based on EMD strategy better compared to time domain method and time-frequency method or not? All these have not been researched yet and we investigate them in our research.

EMD has been widely used for seizure prediction and detection, but for emotion recognition based on EMD, there is not so much research. Higher order statistics of IMFs [30], geometrical properties of the decomposed IMF in complex plane [31], and the variation and fluctuation of IMF [32] are used as features for seizure prediction and detection. For emotion recognition, Mert and Akan extracted entropy, power, power spectral density, correlation, and asymmetry of IMF as features and then utilized independent component analysis (ICA) to reduce dimension of the feature set [33]. The classification accuracy is computed with all the subjects mixed together.

In this paper, we present an emotion recognition method based on EMD. We utilize the first difference of IMF time series, the first difference of the IMF's phase, and the normalized energy of IMF as features. The motivation of using these three features is that they depict the characteristics of IMF in time, frequency, and energy domain, providing multidimensional information. The first difference of time series depicts the intensity of signal change in time domain. The first difference of phase measures the change intensity in phase and normalized energy describes the weight of current oscillation component. The three features constitute a feature vector, which is fed into SVM classifier for emotional state detection.

The proposed method is studied on a publicly available emotional database DEAP [20]. The effectiveness of the three features is investigated. IMF reduction and channel reduction for feature extraction are both discussed, which aim at improving the classification accuracy with less computation complexity. The performance is compared with some other techniques, including fractal dimension (FD), sample entropy, differential entropy, and time-frequency analysis DWT.

## 2. Method

**Figure 1:**
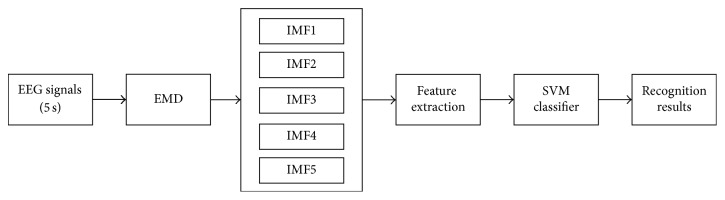
Block diagram of the proposed method.

To realize emotional state recognition, the EEG signals are decomposed into IMFs by EMD. Three features of IMFs, the fluctuation of the phase, the fluctuation of the time series, and the normalized energy, are formed as a feature vector, which is fed into SVM for classification. The whole process of the algorithm is shown in Figure 1.

### 2.1. Data and Materials

DEAP is a publicly available dataset for emotion analysis, which recorded EEG and peripheral physiological signals of 32 participants as they watched 40 music videos. All the music video clips last for 1 minute, representing different emotion visual stimuli, with grade from 1 to 9. Among the 40 music videos, 20 are high valence visual stimuli and 20 are low valence visual stimuli. The situation is exactly the same for arousal dimension. After watching the music video, participants performed a self-assessment of their levels on arousal, valence, liking, dominance, and familiarity, with ratings from 1 to 9. EEG was recorded with 32 electrodes, placing according to the international 10-20 system. Each electrode recorded 63 s EEG signal, with 3 s baseline signal before the trial.

In this paper, we used the preprocessed EEG data for study, with sample rate 128 Hz and band range 4–45 Hz. EOG artefacts were removed as method in [20]. The data was segmented into 60-second trials and a 3-second pretrial baseline removed. The binary classifications of valence and arousal dimension are considered. We utilized the participants' self-assessment as label. If the participant's rating was <5, the label of valence/arousal is low and if the rating was ≥5, the label of valence/arousal is high.

Each music video lasts for 1 minute, and 5 s EEG signals are extracted as a sample. So for each subject who watched 40 music videos, we acquire 480 labeled samples.

### 2.2. Empirical Mode Decomposition

EMD decomposes EEG signals into a set of IMFs by an automatic shifting process. Each IMF represents different frequency components of original signals and should satisfy two conditions: (1) during the whole data set, the number of extreme points and the number of zero crossings must be either equal or differ at most by one; (2) at each point, the mean value calculated from the upper and lower envelope must be zero [29]. For input signal *x*(*t*), the process of EMD is as follows:

(1) Set *h*(*t*) = *x*(*t*) and *h*_old_(*t*) = *h*(*t*).
(2) Get local maximum and minimum of *h*_old_(*t*).
(3) Interpolate the local maximum and minimum with cubic spline function and get the upper envelope *e*_max_(*t*) and lower envelope *e*_min_(*t*).
(4) Calculate the mean value of the upper and lower envelope as
  (1) $$\begin{array}{c} m\left(\;t\;\right)=\frac{\left(e_{min}\left(t\right)+e_{max}\left(t\right)\right)}{2}. \end{array}$$
(5) Subtract *h*_old_(*t*) with *m*(*t*):
  (2) $$\begin{array}{c} h_{new}\left(\;t\;\right)=h_{old}\left(\;t\;\right)-m\left(\;t\;\right). \end{array}$$

If *h*_new_(*t*) satisfies the two conditions of IMF, then the first IMF component imf_1_ is gotten; otherwise, set *h*_old_(*t*) = *h*_new_(*t*) and go to step (2), repeating steps (2)–(5) until *h*_new_(*t*) satisfies the two conditions of IMF. Finally imf_1_ is gotten as

(3) $$\begin{array}{c} imf_{1}=h_{new}\left(\;t\;\right). \end{array}$$

(6) If imf_*n*_ is gotten, set *h*_old_(*t*) as
  (4) $$\begin{array}{c} h_{old}\left(\;t\;\right)=h_{old}\left(\;t\;\right)-imf_{n}. \end{array}$$

Go to step (2) and repeat steps (2)–(5) to get imf_*n*+1_.

By the iterative process described above, *x*(*t*) can be finally expressed as

(5) $$\begin{array}{c} x\left(\;t\;\right)=\overset{L}{\underset{n=1}{\sum }}imf_{n}+r. \end{array}$$

**Figure 2:**
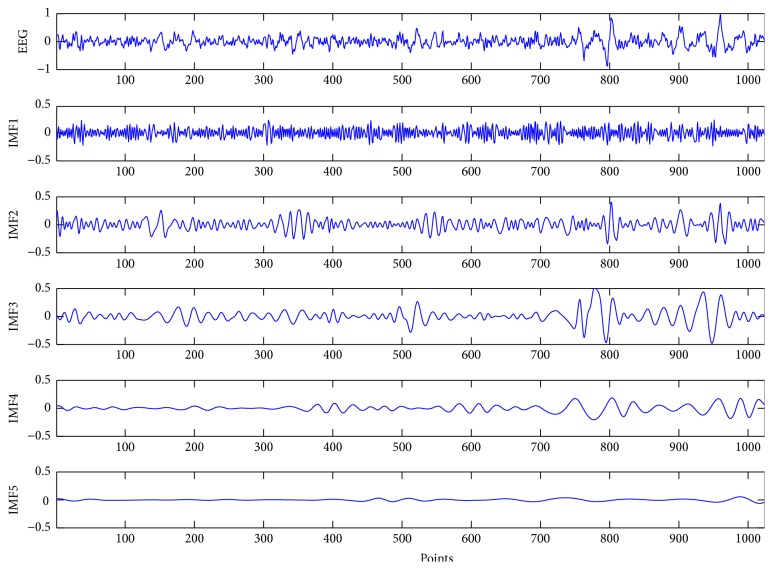
EEG signals and the corresponding first five IMFs.

It is a linear combination of IMF components and the residual part *r*.  Figure 2 shows a segment of original EEG signals corresponding to the first five decomposed IMFs. EMD works like an adaptive high pass filter. It shifts out the fastest changing component first and as the level of IMF increases, the oscillation of IMF becomes smoother. Each component is band-limited, which can reflect the characteristic of instantaneous frequency.

### 2.3. Feature Extraction

In this paper, three features of IMF are utilized for emotion recognition, the first difference of time series, the first difference of phase, and the normalized energy. The first difference of time series depicts the intensity of signal change in time domain. The first difference of phase reveals the change intensity of phase, representing the physical meaning of instantaneous frequency. Normalized energy describes the weight of current oscillation component. The motivation of using these three features is that they depict the characteristics of IMF in time, frequency, and energy domain, utilizing multidimensional information.

#### 2.3.1. First Difference of IMF Time Series

The first difference of times series *D*_*t*_ depicts the intensity of signal change in time domain. Previous research has revealed that the variation of EEG time series can reflect different emotion states [2]. For an IMF component with *N* points, IMF{imf_1_, imf_2_,…, imf_*N*_}, the definition of *D*_*t*_ is

(6) $$\begin{array}{c} D_{t}=\frac{1}{N-1}\overset{N-1}{\underset{n=1}{\sum }}\left|\;imf\left(\;n+1\;\right)-imf\left(\;n\;\right)\;\right|. \end{array}$$

#### 2.3.2. First Difference of IMF's Phase

Based on EMD, EEG is decomposed into multilevel IMFs, each IMF being band-limited and representing an oscillation component of original EEG signals. For an *N*-point IMF, IMF{imf_1_, imf_2_,…, imf_*N*_}, Hilbert transform is applied to it, obtaining an analytic signal *z*(*n*)

(7) $$\begin{array}{c} z\left(\;n\;\right)=x\left(\;n\;\right)+jy\left(\;n\;\right). \end{array}$$

The analytic signal can be further expressed as follows:

(8) $$\begin{array}{c} z\left(\;n\;\right)=A\left(\;n\;\right)e^{j\varphi \left(n\right)}, \end{array}$$

where $A(n)=\sqrt{x(n{)}^{2}+y(n{)}^{2}}$ is the amplitude of *z*(*n*) and *φ*(*n*) = arctan(*y*(*n*)/*x*(*n*)) is the instantaneous phase.

First difference of phase *D*_*p*_ is defined as

(9) $$\begin{array}{c} D_{p}=\frac{1}{N-1}\overset{N-1}{\underset{n=1}{\sum }}\left|\;\varphi \left(\;n+1\;\right)-\varphi \left(\;n\;\right)\;\right| \end{array}$$

which measures the change intensity in phase and represents the physical meaning of instantaneous frequency.

#### 2.3.3. Normalized Energy of IMF

For an *N*-point IMF, IMF{imf_1_, imf_2_,…, imf_*N*_}, the normalized energy *E*_norm_ is defined as follows:

(10) $$\begin{array}{c} E_{norm}=\frac{\sum_{n=1}^{N}imf^{2}\left(n\right)}{\sum_{n=1}^{N}s^{2}\left(n\right)}, \end{array}$$

where *s*(*n*) is the original EEG signal points. So the numerator is the energy of IMF and the denominator represents the energy of original EEG data set. The normalized energy describes the weight of current oscillation component. When fed into the classifier, log⁡(*E*_norm_) is taken as an element of the feature vector according to [26].

### 2.4. SVM Classifier

The extracted features are fed into SVM for classification. SVM is widely used for emotion recognition [34, 35], which has promising property in many fields. In our study, LIBSVM is implemented for SVM classifier with radial basis kernel function and default parameters setting [36].

## 3. Performance Verification

In the following subsections, we test our method on DEAP emotional dataset. Training and classifying tasks were conducted for each subject independently and we utilized leave-one-trail-out validation to evaluate the classification performance. Each subject watched 40 music video clips, and every video clips lasted 1 minute. In our experiment, we utilized the participants' self-assessment as label. Every 5 s EEG signals are extracted as a sample, so for each subject we acquire 480 labeled samples.

In leave-one-trail-out validation, for each subject, 468 samples extracted from 39 trails were assigned to training set, and 12 samples extracted from the remaining one trail were assigned to test set. So there was no correlation between samples in the training set and the test set. Among the total 40 trails of one subject, each trail will be assigned to the test set once as the validation data. The 40 results from the 40 test trails then can be averaged to produce a general estimation for each subject. The final mean accuracy is computed among all the subjects.

### 3.1. Effectiveness of the Features for Emotion Recognition

In order to evaluate the effectiveness of the three features for emotion recognition, we first use only one single feature for classification each time. All the experiments in this subsection are under the condition that the first five IMF components and total 32 electrodes are utilized for feature extraction. The training and classifying for each subject were conducted, respectively, and the mean accuracy was computed among all the subjects.

**Figure 3:**
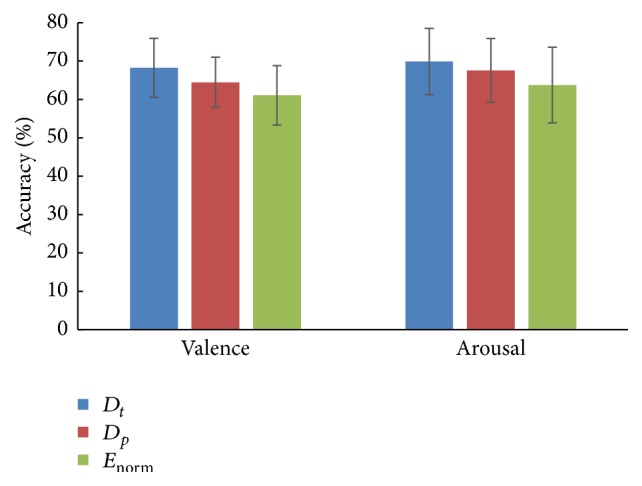
Classification accuracies of three single features. For each subject, one single feature was extracted from the first five IMF components. “*D*_*t*_,” “*D*_*p*_,” and “*E*_norm_” in the figure are corresponding to the three single features, respectively. The mean accuracies for all circumstances were computed among all the subjects. Error bars show the standard deviation of the mean accuracies across all subjects.

The mean classification accuracies of three features are given in Figure 3. It shows that all the three features can distinguish high level from low level on both valence and arousal dimension, higher than random probability of 50%. For valence dimension, the classification accuracy yields 68.27%, 64.46%, and 61.07% with features *D*_*t*_, *D*_*p*_, and *E*_norm_, respectively. For arousal dimension, the classification accuracy yields 69.89%, 67.56%, and 63.76% with features *D*_*t*_, *D*_*p*_, and *E*_norm_, respectively.

### 3.2. IMF Reduction for Feature Extraction

In this subsection, we did two experiments to investigate the role of different IMF components in emotion recognition. In the first experiment, each time only one IMF component was utilized for feature extraction and we analyzed which IMF is effective for emotion recognition. In the second experiment, we further verified whether the combination of multi-IMFs would improve the accuracy.

Table 2 gives all the results in detail. Standard deviation of the mean accuracies across all subjects is shown in parenthesis. “IMF1,” “IMF2,” “IMF3,” “IMF4,” and “IMF5” are corresponding to single IMF component. “IMF1–3” in the table represents the first three IMFs, corresponding to IMF1, IMF2, and IMF3. Similarly, “IMF1–4” and “IMF1–5” are corresponding to the first four IMFs and the first five IMFs, respectively.

It shows that IMF1 yields the best performance, 70.41% for valence and 72.10% for arousal. As the level increases, the performance decreases sharply. The performance of IMF5 is only 55.74% for valence and 62.38% for arousal. We applied *t*-test (*α* < 0.05) to examine the performance between only IMF1 utilized for feature extraction and other circumstances. The null hypothesis is “the performance is similar” and if *p* value is larger than *α*, the null hypothesis is accepted. The results of *t*-test in Table 1 show that the performance of IMF1 is more splendid than other single components, IMF2, IMF3, IM4, and IMF5, with *p* far less than 0.05. It also shows that performance of multi-IMF combinations is similar to only IMF1 utilized for feature extraction, with *p* larger than 0.05.

IMF1 represents the fastest changing component of EEG signals, with the highest frequency characteristic. As the level increases, the oscillation becomes smoother with frequency becoming lower and lower. So we infer that the valence and arousal of emotion relate more tightly to high frequency. It is also coincided with the finding in [26] that Beta (16–32 Hz) and Gamma (32–64 Hz) bands are successfully selected more often than other bands. These two bands are higher frequency subbands of EEG signals.

So combining the results of classification accuracy and *t*-test, in practical use, we just need to extract features from IMF1, which will save vast time and relieve computation burden because only one level of EMD decomposition needed to be done.

### 3.3. Channel Reduction for Feature Extraction

Form verification in Section 3.2, we know that using component IMF1 will achieve good performance. In this subsection, we will investigate which electrodes are informative based on EMD strategy.

Fisher distance is an efficient criterion of divisibility between two classes, which is broadly used in pattern recognition. It computes the ratio of between-class scatter degree and within-class scatter degree between two classes. Larger ratio means larger divisibility of the two classes. In our experiment, we used fisher distance to mark important electrodes under condition that IMF1 is used for feature extraction. For each channel, fisher distance is calculated among features extracted from one subject's total 480 labeled emotion samples.

**Figure 4:**
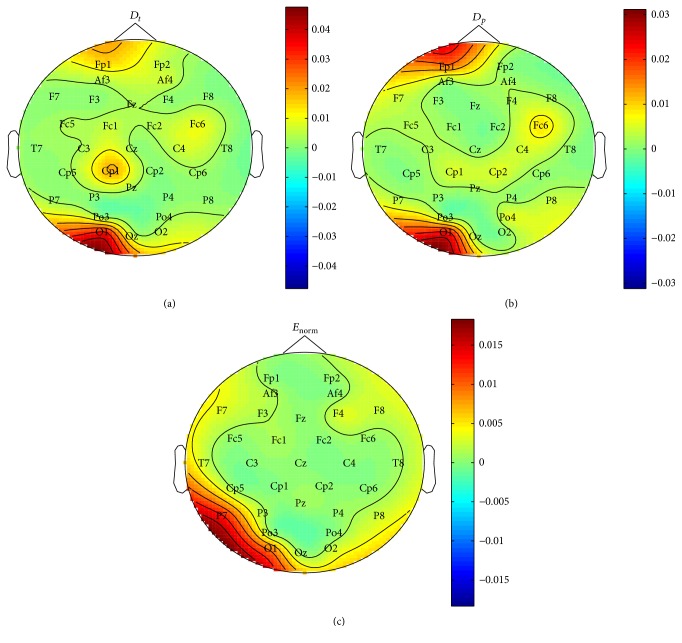
Fisher distance of different channels with subject 1. Features are extracted from component IMF1. For each channel, Fisher distance is calculated among features extracted from 480 labeled emotion samples of subject 1. (a) Fisher distance under feature *D*_*t*_. (b) Fisher distance under feature *D*_*p*_. (c) Fisher distance under feature *E*_norm_.

Figure 4 gives fisher distance on valence dimension with subject 1.  Figure 4(a) shows that, under feature *D*_*t*_, electrodes Fp1, Fp2, FC6, Cp1, O1, and Oz have larger values.  Figure 4(b) shows that, under feature *D*_*p*_, Fp1, FC6, Cp1, Cp2, O1, Oz, P7, and P8 have larger values.  Figure 4(c) shows that, under feature *E*_norm_, F7, F8, T7, T8, P7, P8, O1, O2, and Oz have larger values.

Based on the analysis of all the subjects, we selected the following 8 electrodes Fp1, Fp2, F7, F8, T7, T8, P7, and P8 for channel reduction verification. Table 2 gives *F*1 score and classification accuracy with 8 channels selected for emotion recognition. We see that *F*1 score is 0.7374 for valence and 0.7769 for arousal. The classification accuracy with 8 channels is 69.10% for valence and 71.99% for arousal, slightly lower than accuracy with total 32 channels. We also applied *t*-test to examine whether the performance of 8 channels is similar to total 32 channels. The null hypothesis is “the performance is similar” and if *p* value is larger than *α*, the null hypothesis is accepted. The *t*-test result shows that the performance under 8 channels and 32 channels is similar, with *p* = 0.4194 for valence and *p* = 0.9521 for arousal.

So in practical use, we just need to extract features from IMF1 with 8 channels. Our offline experiment used every 5 s EEG signals as a labeled emotion sample. This infers that our method may provide a new solution for real-time emotion recognition in BCI systems.

### 3.4. Results Comparison with Other Methods

In this subsection, we compared our proposed method with some classical methods, including fractal dimension (FD), sample entropy, differential entropy, and time-frequency analysis DWT. We used box counting for fractal dimension calculating. The parameter for sample entropy SampEn(*m*, *r*, *N*) was set as *r* = 0.2, *m* = 2, and *N* = 128. We used “db4” decomposition to realize DWT. Then the differential entropy of Beta (16–32 Hz) and Gamma (32–64 Hz) bands is extracted as features. Our method used IMF1 for feature extraction of *D*_*t*_, *D*_*p*_, and *E*_norm_. For all the methods, 8 selected channels FP1, FP2, F7, F8, T7, T8, P7, and P8 are used for feature extraction.

**Figure 5:**
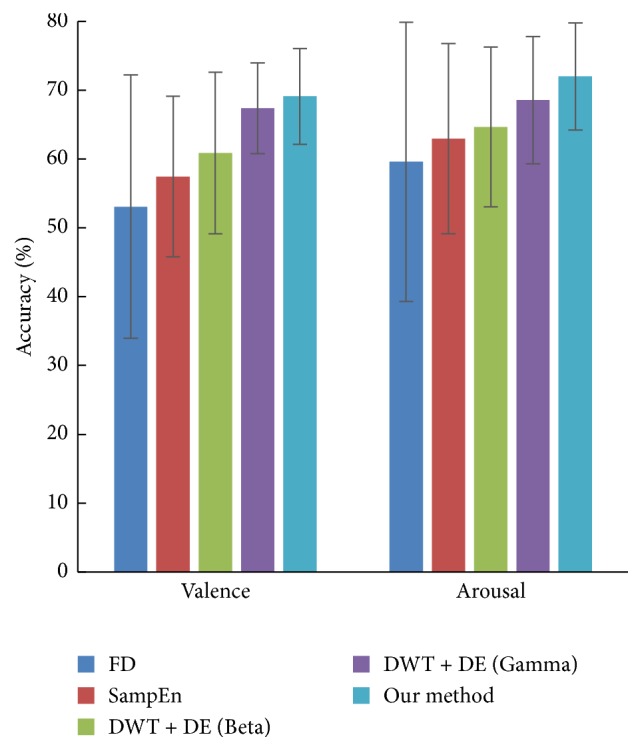
Classification accuracies of different methods. “FD,” “SampEn,” and “DE” in the figure are corresponding to fractal dimension, sample entropy, and differential entropy, respectively. The mean accuracy was computed among all the subjects. Error bars show the standard deviation of the mean accuracies across all subjects.

From Figure 5 and Table 3, we see that our method yields the highest accuracy, 69.10% for valence and 71.99% for arousal. We applied *t*-test (*α* < 0.05) to examine the performance between classical method and our method. The null hypothesis is “the performance is similar” and if *p* value is larger than *α*, the null hypothesis is accepted. The results of *t*-test in Table 3 show that the performance of our method is more splendid than fractal dimension, sample entropy, and differential entropy of Beta band with *p* far less than 0.05. It also shows that the performance of our method is similar and better than the differential entropy of Gamma band.

EMD strategy outperforms time domain method, including fractal dimension and sample entropy. This is because compared to methods in time domain, EMD has the advantage of utilizing more oscillation information. Compared to time-frequency method DWT, EMD can decompose EEG signals automatically, getting rid of selecting transform window first. The classification accuracy is also higher than DWT. So the experiment results infer that our method based on EMD strategy is suitable for emotion recognition from EEG signals.

## 4. Discussion

Emotion recognition from EEG signals has achieved significant progress in recent years. Previous methods are usually conducted in time domain, frequency domain, and time-frequency domain. In this paper, we propose a method of feature extraction for emotion recognition in EMD domain, a new aspect of view. By utilizing EMD, EEG signals can be decomposed into different oscillation components named IMF automatically. The characteristics of IMF are utilized as features for emotion recognition, including the first difference of time series, the first difference of phase, and the normalized energy.

Compared to methods in time domain, EMD has the advantage of utilizing more frequency information. The experiment results show that the proposed method outperforms method in time domain, such as fractal dimension in [3, 4] and sample entropy in [5]. Compared to time-frequency methods, such as STFT and DWT, EMD can decompose EEG signals automatically, getting rid of selecting transform window first. The classification accuracy is also higher than DWT in [18].

We investigate the role of each IMF in emotion classification. Features extracted from IMF1 yield the highest accuracy. IMF1 is corresponding to the fastest changing component of EEG signals, so our study confirms the deduction that emotion is more relative to high frequency component. This consists with findings in [26] that Beta (16–32 Hz) and Gamma (32–64 Hz) bands are successfully selected more often than other bands.

Finally, we selected 8 informative channels based on EMD strategy, namely, FP1, FP2, F7, F8, T7, T8, P7, and P8. Our proposed method just needs to extract features from IMF1 with 8 channels, which will save time and relieve computation burden. Also in our experiment, every 5 s EEG signals are extracted as a sample, so it may provide a new solution for real-time emotion recognition in BCI systems.

Our limitation is that now we just test it on DEAP dataset, so in the future we want to experiment it on more emotional datasets to verify the method comprehensively. Also we will utilize more strategies such as feature smoothing and deep network to improve the classification accuracy.

## 5. Conclusion

In this paper, an emotion recognition method based on EMD using three statistics is proposed. An extensive analysis has been carried out to investigate the effectiveness of the features for emotion classification. The results show that the three features are suitable for emotion recognition. Then the effect of each IMF component is inquired. The results reveal that, among the multilevel IMFs, the first component IMF1 plays the most important role in emotion recognition. Also the informative channels based on EMD strategy are investigated and 8 channels, namely, FP1, FP2, F7, F8, T7, T8, P7, and P8, are selected for feature extraction. Finally, the proposed method is compared with some classical methods and our method yields the highest accuracy.

## Figures and Tables

**Table 1:** Comparison of performance for different IMFs selected for feature extraction (32 channels) (standard deviation shown in parentheses).

Component	Valence		Arousal	
	Accuracy (%)	*t*-test (IMF1)	Accuracy (%)	*t*-test (IMF1)
IMF1	**70.41** (7.05)	*p* = 1	**72.10** (7.51)	*p* = 1
IMF2	63.47 (7.10)	*p* = 0.0002	66.58 (9.36)	*p* = 0.0032
IMF3	61.45 (8.57)	*p* = 0	64.56 (10.52)	*p* = 0.0019
IMF4	59.55 (8.56)	*p* = 0	63.99 (10.96)	*p* = 0.0012
IMF5	55.74 (9.20)	*p* = 0	62.38 (12.23)	*p* = 0
IMF1-2	69.02 (7.00)	*p* = 0.4399	70.47 (8.29)	*p* = 0.1940
IMF1–3	68.47 (6.69)	*p* = 0.2705	70.08 (8.10)	*p* = 0.3116
IMF1–4	67.99 (6.58)	*p* = 0.1688	69.60 (8.08)	*p* = 0.2107
IMF1–5	67.59 (6.58)	*p* = 0.1086	69.00 (8.37)	*p* = 0.1293

**Table 2:** Performance of 8 channels selected for feature extraction (Fp1, Fp2, F7, F8, T7, T8, P7, and P8) (standard deviation shown in parentheses).

Predict	Label			
	Valence		Arousal	
	High	Low	High	Low
High	6664	2723	7493	2748
Low	2024	3949	1555	3564
*F*1 score	0.7374		0.7769	
Accuracy (%)	69.10 (6.95)		71.99 (7.77)	

**Table 3:** The mean accuracy of different kinds of methods (Fp1, Fp2, F7, F8, T7, T8, P7, and P8) (standard deviation shown in parentheses; statistical analysis shown in column *t*-test).

Methods	Valence		Arousal	
	Accuracy (%)	*t*-test (our method)	Accuracy (%)	*t*-test (our method)
Fractal dimension	53.08 (19.14)	*p* = 0	59.61 (20.28)	*p* = 0.0034
Sample entropy	57.44 (11.66)	*p* = 0	62.96 (13.82)	*p* = 0.0024
DWT + differential entropy (Beta)	60.87 (11.74)	*p* = 0.0013	64.66 (11.59)	*p* = 0.0048
DWT + differential entropy (Gamma)	67.36 (6.61)	*p* = 0.3185	68.55 (9.28)	*p* = 0.1189
Our method	69.10 (6.95)	*p* = 1	71.99 (7.77)	*p* = 1
